# A Computational Study of Blood Flow Dynamics in the Pulmonary Arteries

**DOI:** 10.1007/s10013-022-00595-y

**Published:** 2022-12-14

**Authors:** Fabio Marcinno’, Alberto Zingaro, Ivan Fumagalli, Luca Dede’, Christian Vergara

**Affiliations:** 1 Dipartimento di Chimica, Materiali e Ingegneria Chimica “Giulio Natta”, Politecnico di Milano, Piazza Leonardo da Vinci 32, Milan, 20133 Italy; 2 MOX, Dipartimento di Matematica, Politecnico di Milano, Piazza Leonardo da Vinci 32, Milan, 20133 Italy

**Keywords:** Pulmonary circulation, Hemodynamics, Computational fluid dynamics, Geometric multiscale model, Partitioned coupling algorithm, 65M60, 76D05

## Abstract

In this work we study the blood dynamics in the pulmonary arteries by means of a 3D-0D geometric multiscale approach, where a detailed 3D model for the pulmonary arteries is coupled with a lumped parameters (0D) model of the cardiovascular system. We propose to investigate three strategies for the numerical solution of the 3D-0D coupled problem: the Splitting-Explicit and Implicit algorithms, where information are exchanged between 3D and 0D models at each time step at the interfaces, and the One-Way algorithm, where the 0D is solved first off-line. In our numerical experiments performed in a realistic patient-specific 3D domain with a physiologically calibrated 0D model, we discuss first the issue on instabilities that may arise when not suitable connections are considered between 3D and 0D models; second we compare the performance and accuracy of the three proposed numerical strategies. Finally, we report a comparison between a healthy and a hypertensive case, providing a preliminary result highlighting how our method could be used in future for clinical purposes.

## Introduction

The pulmonary arteries are among the largest arteries in human body, and they are located between the right ventricle and the lungs; they carry de-oxygenated blood coming from the venous circulation to the pulmonary alveoli, where it is oxygenated [25]. The study of the pulmonary arteries hemodynamics is fundamental since the pulmonary circulation is exposed to critical diseases. One of the most important is Pulmonary Arterial Hypertension (PAH) which leads to an increased resistance to blood flow in the lungs [9, 16].

Computational methods revealed to be an effective, non-invasive tool for the quantitative description of hemodynamics [20, 31]. One of the most used computational method in hemodynamics is the geometric multiscale approach [32, 33]. In such context, the cardiovascular system is divided in two different parts: the part of interest, which is modeled by means of a high detailed model (for example, the 3D Navier–Stokes equations), and the remaining part, which is modeled by means of a geometrically reduced model such as the lumped parameters one, since a detailed description of the hemodynamics outside the region of interest is not needed. We refer to [4, 10, 11, 24, 26, 27, 46] for other works about geometric multiscale coupling.

In this context, the pulmonary circulation is less studied than the systemic arterial one, but in the recent years its interest is increased specially due to the spreading of the Coronavirus COVID19 disease. In [23], the authors simulate the fluid-structure interaction (FSI) of a healthy pulmonary arterial tree using a segregated approach in which the outlet boundary conditions are imposed by means of the Windkessel model; in [21], a FSI of the pulmonary arteries is proposed where traction-free conditions are prescribed at the outlet; in [40], the computational fluid dynamics (CFD) of the pulmonary arteries is simulated under resting and exercise conditions and the outlet boundary conditions are imposed by means of a pure resistance lumped parameter; in [45], the authors simulate FSI in the pulmonary arteries and vary the vessel wall stiffness to simulate different PAH scenarios, with outlet boundary conditions imposed by means of the Windkessel model.

About 3D-0D geometric multiscale modelling in the context of pulmonary circulation, recent works focused on the coupling with a closed-loop 0D model to study specific unhealthy scenarios [8, 28, 38]. For example, in [8], the authors studied the hybrid Norwood procedure, whereas in [28] the authors studied the Potts shunt as a potential palliative treatment for suprasystemic idiopathic PAH. It is worth also citing papers on the 3D-0D geometric multiscale modelling in the context of PAH [29, 39, 43–45].

In the present work, we investigate the coupling between a 3D fluid-dynamic model of the pulmonary arteries and a closed-loop lumped parameters model accounting for the whole cardiovascular system and we compare different numerical algorithms and scenarios. The closed-loop 0D model brings to a multiscale 3D-0D problem allowing us to impose physiological conditions to the 3D domain. In particular, we study the reliability of including a RLC model downstream the diode representing the pulmonary artery in order to prevent numerical instabilities. We consider three different numerical algorithms for the solution of the 3D-0D problem, a Splitting-Explicit coupled algorithm, a Splitting-Implicit coupled algorithm [26, 33] and a One-Way decoupled algorithm [8] the results are compared in terms of hemodynamic variables (velocity, pressure and wall shear stress). Finally, we report a comparison between the healthy and the simulated PAH cases.

The paper outline is as follows. Section 2 is dedicated to the mathematical model of the geometric multiscale coupling. In Section 3 the numerical algorithms are described. Finally, in Section 4 we report the numerical results aiming at showing the reliability of the proposed model, together with a discussion of possible instabilities which may arise in specific conditions.

## The 3D-0D Geometric Multiscale Model

**Fig. 1:**
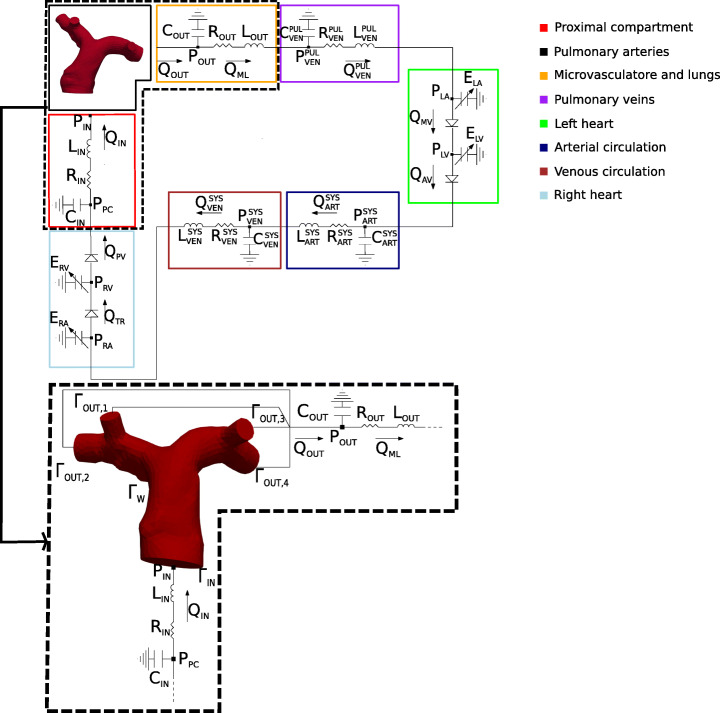
Top: Geometric multiscale model of the entire cardiovascular system obtained by the coupling between the 3D pulmonary artery and the Open-0D model. Bottom: Zoom on the region where the 3D-0D coupling occurs. In the squares the four interface variables

In medium and large vessels as the pulmonary arteries, blood is well modeled as an incompressible, homogeneous and Newtonian fluid [3, 6, 31]. Thus, we consider the 3D incompressible Navier–Stokes equations, where $\boldsymbol {u}(\boldsymbol {x},t):{{\varOmega }}\times \mathbb {R}^{+} \to \mathbb {R}^{3}$ is the blood velocity, $p(\boldsymbol {x},t):{{\varOmega }} \times \mathbb {R}^{+} \to \mathbb {R}$ the blood pressure, *μ* stands for the dynamic blood viscosity, *ρ* is the blood density and ***n*** is the outgoing normal vector from the boundaries. In [23], it has been demonstrated that the rigid wall assumption is able - in first approximation - to well approximate the results obtained by a FSI simulation for the largest branches of the pulmonary arteries since the compliance is not so relevant due to the small displacements featured by our cases (notice that also in the hypertension case the pressure is below 35 mmHg, see below). Accordingly, in this work we consider rigid walls. Referring to Fig. 1, the 3D computational domain is ${{\varOmega }} \subset \mathbb {R}^{3}$, where *Γ*_*IN*_ is the inlet boundary, *Γ*_*OUT*,*i*_ (with $i=1,\dots ,4$) are the outlet boundaries and *Γ*_*W*_ is the vessel wall.

The fluid dynamics of the remaining part of the cardiovascular system is modeled by means of a lumped parameters 0D model based on electrical analogies [33]. In particular, the voltage and the current represent the pressure and the blood flow, respectively; the resistance corresponds to the effect of the blood viscosity, the capacity the wall compliance, whereas the inductance the inertial effects of the blood flow. The four cardiac valves are modeled by means of non-ideal diodes, and the heart is described with time dependent elastances representing the pump function [12, 33]. We refer to [36] for the complete list of the differential-algebraic equations of the lumped parameters model. Moreover, we define ***y*** as the vector of the state variables and ***z*** as the vector of the algebraic variables.

The 3D and 0D models are coupled through suitable interface conditions (I.C.) at the inlet and outlet boundaries guaranteeing the continuity of flow rates and pressures. We refer to the lumped parameter model used here as *Open-0D model*, since it needs to be closed with the 3D model. It is worth noting that the information coming from the 0D model is called *defective* since it prescribes only one scalar function of time over the entire boundary of the 3D domain, thus representing an incomplete information for the 3D formulation [13, 14, 30]. In this work, due to laminar assumption of blood flow that holds true in the pulmonary artery [17, 18], defective flow rate information is completed by means of the prescription of a parabolic velocity profile. Regarding the defective mean pressure condition, a constant and normal pressure is instead prescribed according to the do-nothing approach, see [15].

Thus, let us define *T* as the final time of the simulation, therefore the strong formulation of the geometric multiscale 3D-0D model reads as follows:

Find ***u***, *p* and ***y***, ***z***, for any *t* ∈ (0,*T*], such that

$$ \left\{\begin{array}{ll} 3D~~\left\{\begin{array}{ll} \rho \frac{\partial\boldsymbol{u}}{\partial t} + \rho (\boldsymbol{u} \cdot \nabla)\boldsymbol{u} - \nabla \cdot \boldsymbol{T}(\boldsymbol{u},p) = \boldsymbol{0} & \quad \text{in} \quad {{\varOmega}},\\ \nabla\cdot\boldsymbol{u}=0 & \quad \text{in} \quad {{\varOmega}},\\ \boldsymbol{u}(\boldsymbol{x},0) = \boldsymbol{0} & \quad \text{in} \quad {{\varOmega}},\\ \boldsymbol{u}(\boldsymbol{x},t) = \boldsymbol{0} & \quad \text{on} \quad {{\varGamma}}_{W}, \end{array}\right.\\ I.C.~\left\{\begin{array}{l} \displaystyle Q_{IN} = {\int}_{{{\varGamma}}_{{IN}}}\boldsymbol{u}\cdot \boldsymbol{n} d{{\varGamma}},\\ \displaystyle P_{IN} = \frac{1}{|{{\varGamma}}_{IN}|}{\int}_{{{\varGamma}}_{IN}} \boldsymbol{T}(\boldsymbol{u},p)\boldsymbol{n}\cdot\boldsymbol{n} d{{\varGamma}}, \\ \displaystyle Q_{OUT} = {\int}_{{{\varGamma}}_{OUT}}\boldsymbol{u}\cdot \boldsymbol{n} d{{\varGamma}},\\ \displaystyle P_{OUT} = \frac{1}{|{{\varGamma}}_{OUT}|}{\int}_{{{\varGamma}}_{OUT}} \boldsymbol{T}(\boldsymbol{u},p)\boldsymbol{n}\cdot\boldsymbol{n} d{{\varGamma}}, \end{array}\right.\\ Open-0D~\left\{\begin{array}{l} \frac{d}{dt}\boldsymbol{y}={f^{O}_{1}}(t,\boldsymbol{y},\boldsymbol{z},P_{IN},Q_{OUT}),\\ \boldsymbol{y}(0)=\boldsymbol{y}_{0},\\ \boldsymbol{z} = {f^{O}_{2}}(t,\boldsymbol{y}), \end{array}\right. \end{array}\right. $$

where ***T*** = −*p****I*** + *μ*(∇***u*** + (∇***u***)^*T*^), ${{\varGamma }}_{OUT}={\sum }_{i=1}^{4}{{\varGamma }}_{OUT,i}$, ${f^{O}_{1}}$ and ${f^{O}_{2}}$ are the right hand side terms of the differential and algebraic equations of the Open-0D model. It is worth reporting the equations describing the heart chambers and the cardiac valves; in particular the elastance *E* has the structure *E*(*t*) = *E*_*a*_*r*(*t*) + *E*_*b*_ [5], with

$$ r(t) = \left\{\begin{array}{ll} \frac{1}{2}\left( 1-\cos\left( \frac{\pi t}{T_{contr}}\right)\right), &\quad t\leq T_{contr}, \\ \frac{1}{2}\left( 1+\cos\left( \frac{\pi (t-T_{contr})}{T_{relax}}\right)\right), &\quad T_{contr}<t\leq T_{contr}+T_{relax}, \\ 0, &\quad t>T_{contr}+T_{relax}, \end{array}\right. $$

where *E*_*a*_ and *E*_*b*_ are the active and passive elastances, respectively, *T*_*contr*_ is the duration of the chamber contraction and *T*_*relax*_ is the duration of the chamber relaxation. The resistance of the cardiac valves is in general defined as follows:

$$ \begin{array}{@{}rcl@{}} R = 10^{c}, \quad c &=& \log_{10}R_{\min} + \left( \log_{10}R_{\max} - \log_{10}R_{\min}\right)\\ &&\times\left[\frac{1}{2} + \frac{1}{\pi}\arctan\left( \frac{200\pi}{2}(P_{2} - P_{1})\right)\right], \end{array} $$

where $R_{\max \limits }$ is the resistance when the valve is closed and $R_{\min \limits }$ corresponds to the resistance when the valve is open, and *P*_1_ and *P*_2_ are the pressures upstream and downstream the valve, respectively.

In Fig. 1, we report the representation of the geometric multiscale model highlighting the zones of interest (the resistance of the cardiac valves is omitted).

Notice from Fig. 1 that we couple the 3D model not directly with the pulmonary valve (diode, light blue block) in the upstream region. Instead, we introduce an additional block, denoted as “proximal compartment” and colored in red in Fig. 1, representing the proximal part of the pulmonary artery. We couple the inlet of the 3D model to the proximal compartment, and we found that this coupling choice preserves the appearance of instabilities, as we better discuss in Section 4.3. Moreover, as first approximation, we couple the outlets of the pulmonary arteries with a single compartment (representing the arterial pulmonary microvascolature); therefore, the downstream pressure is the same at all outlets.

## Algorithms for the Numerical Solution

In order to numerically solve the geometric multiscale model, we introduce a uniform time discretization in which ${{\varDelta }} t = \frac {T}{N_{t}}$ is the step size, and *N*_*t*_ are the number of steps in which the time interval is subdivided. The *n* th temporal time step is defined as *t*^*n*^ = *nΔt* for $n = 0, \dots , N_{t}$. Given a function of time *v*(*t*), we denote by $v^{n}\simeq v(t^{n})$ its approximation after the time discretization.

Concerning the 3D model, the time discretization is obtained by means of the first order backward differentiation formula (implicit Euler) and the convective term is treated with a semi-implicit treatment [34]. The time discretization of the 0D model is achieved by means of the explicit Euler scheme or the 4th order Runge–Kutta explicit method [35], depending on the test, see Section 4.

For the solution of the coupled geometric multiscale problem, we introduce three strategies: the *Splitting-Explicit Algorithm*, the *Splitting-Implicit Algorithm* and the *One-Way Algorithm*, described in what follows.

### The Splitting-Explicit Algorithm

The geometric multiscale coupling is solved first by means of the Splitting-Explicit (SE) algorithm through a partitioned and explicit way; this means that the lumped parameter model and the 3D model are solved sequentially once per time step by means of different numerical solvers through the exchange of information at the interfaces [8, 28].

The SE algorithm is constructed as follows: at time *t*^*n*+ 1^ the 3D model receives from the Open-0D model the flow rate datum $Q^{n}_{IN}$ computed at previous time step imposed at the inlet *Γ*_*IN*_ by means of a parabolic velocity profile, i.e. ***u***^*n*+ 1^ = ***g***, with

$$ {\int}_{{{\varGamma}}_{IN}}\boldsymbol{g}\cdot\boldsymbol{n} d{{\varGamma}} = Q^{n}_{IN},\qquad \boldsymbol{g}(r) = -2\frac{Q^{n}_{IN}}{\pi R^{2}}\left( 1-r^{2}/R^{2}\right)\boldsymbol{n}, $$

where *R* is the radius of the circle located at the inlet of the pulmonary artery and obtained by considering a small flow extension of the reconstructed inlet [1], and *r* is the radial coordinate. Moreover, it receives the mean pressure datum $P_{OUT}^{n}$ imposed at each of the four in parallel outlets *Γ*_*OUT*,*i*_ by means of the do-nothing approach, i.e

$$ \boldsymbol{T}\left( \boldsymbol{u}^{n+1},p^{n+1}\right)\boldsymbol{n} = -P_{OUT}^{n} \boldsymbol{n} \qquad \text{on} \quad {{\varGamma}}_{OUT,i} \times (0,{T}],\qquad i=1,\ldots,4. $$

We use for the 3D fluid dynamics the compact notation *F*(***u***^*n*+ 1^,*p*^*n*+ 1^) = 0 together with the interface boundary conditions involving $Q^{n}_{IN}$ and $P^{n}_{OUT}$, which allows to compute the other two interface data $P_{IN}^{n+1}$ and $Q_{OUT}^{n+1}$. These latter information are passed to the Open-0D model as forcing terms. In particular, we compactly use for the Open-0D model the notation $\boldsymbol {y}^{n+1}={f^{O}_{1}}(t^{n};\boldsymbol {y}^{n},\boldsymbol {z}^{n+1},P_{IN}^{n+1},Q_{OUT}^{n+1})$ and $\boldsymbol {z}^{n+1} ={f^{O}_{2}}(t^{n},\boldsymbol {y}^{n})$ which is solved allowing to compute the quantities $Q_{IN}^{n+1}$ and $P_{OUT}^{n+1}$ for the next time step.

We report the Splitting-Explicit scheme in Algorithm 1.

**Algorithm 1:**
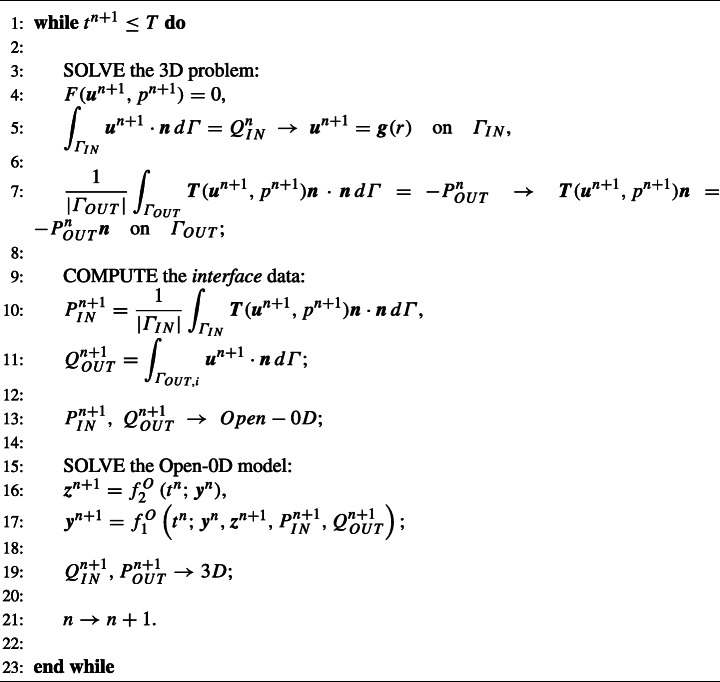
Splitting-explicit algorithm.

### The Splitting-Implicit Algorithm

In the Splitting-Implicit (SI) algorithm, the lumped parameter model and the 3D model are instead solved sequentially many times per time step until convergence of the interface conditions. This corresponds to an implicit time discretization of the coupling that guarantees absolute stability for any value of *Δt*. On the contrary, the Splitting-Explicit algorithm reported above could be seen as an explicit time discretization of the interface conditions, that leads to the same accuracy of the SI algorithm, but with a constraint on the values of *Δt* that guarantee absolute stability.^1^ The SI scheme is reported in Algorithm 2 (notice that in this case we have $\boldsymbol {g}(r) = -2\frac {Q^{n+1,(k-1)}_{IN}}{\pi R^{2}}(1-r^{2}/R^{2})\boldsymbol {n}$).

**Algorithm 2:**
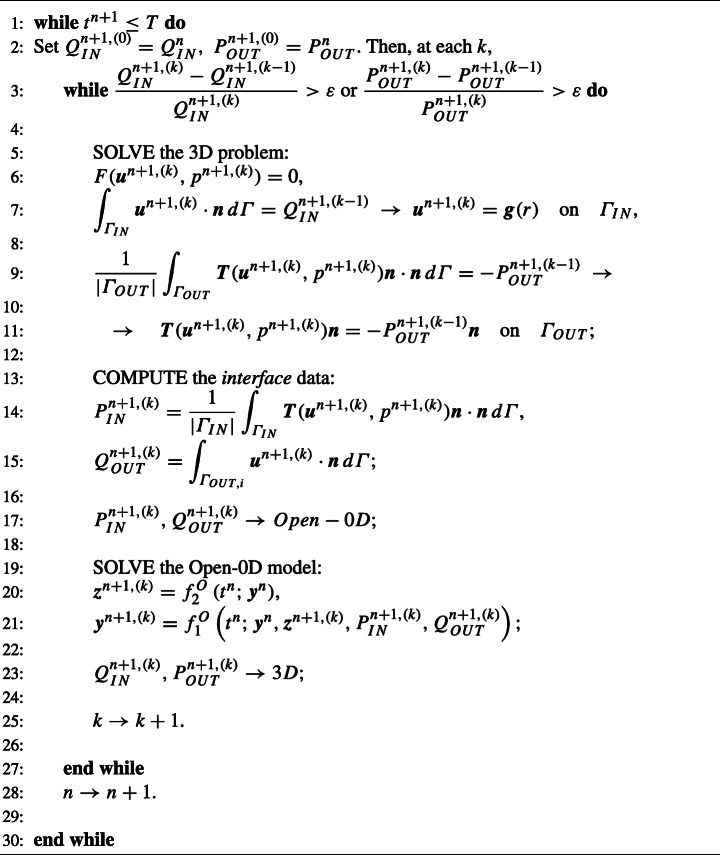
Splitting-implicit algorithm.

### The One-Way Algorithm

**Fig. 2:**
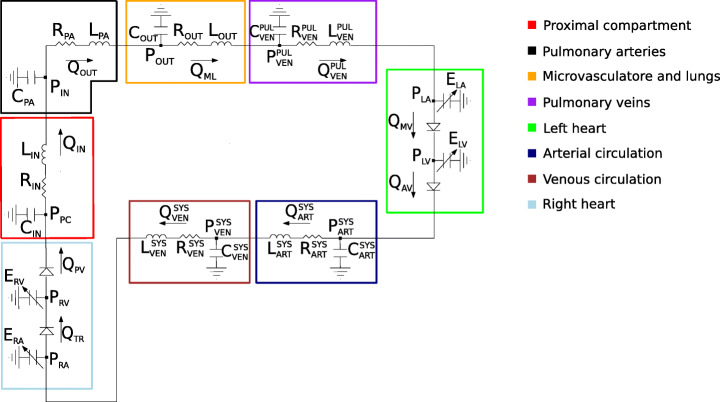
Closed-0D model used in the One-Way Algorithm. *Q*_*IN*_ and *P*_*OUT*_ are provided to the 3D pulmonary arteries (Algorithm 3). Notice the 3D pulmonary arteries compartment, here is replaced by the black RLC network

The One-Way algorithm couples the 3D and the Closed-0D model obtained by the Open-0D model inserting a RLC circuit representing the pulmonary artery (see the black box in Fig. 2).

In this case the coupling is only in one direction; in particular, the Closed-0D model is solved off-line independently. Afterward, at each time step *t*^*n*^ the 0D flow rate $Q^{n}_{IN}$ and the mean pressure $P^{n}_{OUT}$ are passed to the 3D model, without any feedback to the Closed-0D model.

We report the One-Way scheme in Algorithm 3, where ${f^{C}_{1}}$ and ${f^{C}_{2}}$ are the right hand side terms of the differential and algebraic equations of the Closed-0D model.

### Space Discretization

For the solution of the 3D problem in all the algorithms presented in the previous section, we consider the Finite Elements approximation. In particular, we use $\mathbb {Q}$1-$\mathbb {Q}$1 Finite Elements for the approximation of the pressure and each velocity component, introducing $ {X_{1}^{h}}({{\varOmega }}) = \{v^{n+1}_{h} \in C^{0}({{{\varOmega }}}) : v^{n+1}_{h}\in \mathbb {Q}1,~\forall K \in T_{h}\}$, where *T*_*h*_ is partition of the domain into hexahedral cells *K*, together with a stabilization term to ensure uniqueness of the solution given by the PSPG technique. Moreover, to guarantee stability of the numerical solution in presence of a dominated advection regime, we also include the SUPG stabilization [34]. Finally, due to the presence of backflows at the outlets that lead to the production of instabilities due to the lack of energy dissipation of the convective term, we also add a backflow stabilization non-consistent term [2].

**Algorithm 3:**
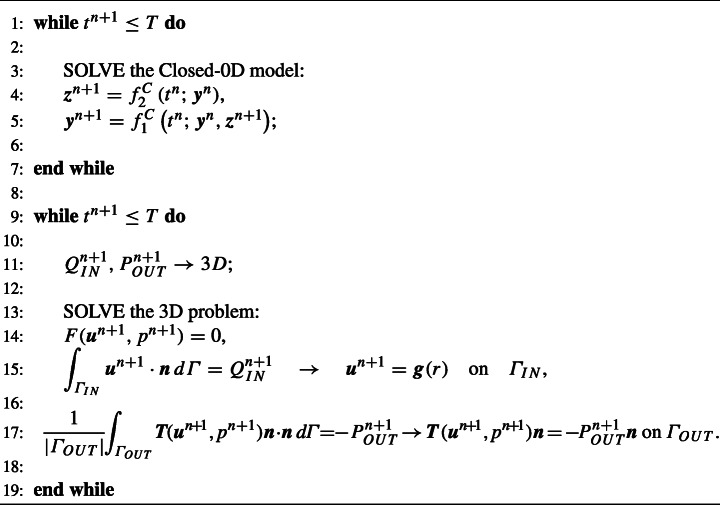
One-way algorithm.

Thus, the fully discretized formulation reads as follows: for every *n* = 0,1,…,*N*_*t*_ − 1, find $\boldsymbol {u}_{h}^{n+1} \in \boldsymbol {V}^{h}$ and $p_{h}^{n+1} \in Q^{h}$ such that

$$ \begin{array}{@{}rcl@{}} \left( \rho\left( \frac{\boldsymbol{u}^{n+1}_{h} -\boldsymbol{u}^{n}_{h}}{{{\varDelta}} t}\right),\boldsymbol{v}_{h}\right) + \left( \rho\boldsymbol{u}_{h}^{n}\cdot \nabla\boldsymbol{u}_{h}^{n+1},\boldsymbol{v}_{h}\right) + \left( \mu (\nabla\boldsymbol{u}_{h}^{n+1} + (\nabla\boldsymbol{u}_{h}^{n+1})^{T},\nabla\boldsymbol{v}_{h}\right) - \\ +\left( p_{h}^{n+1},\nabla \cdot{\boldsymbol{v}_{h}}\right) + {\int}_{{{\varGamma}}_{OUT,i}}\beta \frac{\rho}{2}\left( \boldsymbol{u}_{h}^{n}\cdot\boldsymbol{n}\right)_{-}\boldsymbol{u}_{h}^{n+1}\cdot\boldsymbol{v}_{h} d{{\varGamma}} + s_{h}(\boldsymbol{u}_{h}^{n+1},p_{h}^{n+1};\boldsymbol{v}_{h},q_{h})\\ = {\int}_{{{\varGamma}}_{OUT,i}} P_{OUT}^{n}\boldsymbol{n}\cdot\boldsymbol{v}_{h} d{{\varGamma}} \qquad \forall \boldsymbol{v}_{h} \in \boldsymbol{W}^{h},\\ (\nabla \cdot \boldsymbol{u}_{h}^{n+1},q_{h})= 0 \qquad \forall q_{h} \in Q^{h}, \\ \boldsymbol{u}_{h}^{0} = \boldsymbol{0} \qquad \qquad \text{in }~{{\varOmega}}, \end{array} $$

where $\boldsymbol {V}^{h} = \{\boldsymbol {v}\in [{X_{1}^{h}}({{\varOmega }}) ]^{3} : \boldsymbol {v}_{{{\varGamma }}_{IN}} = \boldsymbol {g}, \boldsymbol {v}_{{{\varGamma }}_{W}} = \boldsymbol {0}\}$, $Q^{h} = {X_{1}^{h}}({{\varOmega }})$, $\boldsymbol {W}^{h} = \{\boldsymbol {v} \in [{X^{h}_{1}}({{\varOmega }})]^{3} : \boldsymbol {v}_{{{\varGamma }}_{IN}\cup {{\varGamma }}_{w}} = \boldsymbol {0}\}$, *β* is the backflow stabilization parameter, and *s*_*h*_ is the SUPG-PSPG stabilization term [41].

## Numerical Results

In this section, we present some numerical results of the proposed computational models to handle the geometric multiscale coupling in the pulmonary arteries. First, we report the results about the comparison between the Splitting-Explicit and the Splitting-Implicit algorithms (Section 4.2) and about the mesh convergence (Section 4.3). Second, we discuss the presence of possible numerical instabilities and how to stabilize the solution (Section 4.4). Then, we report the results obtained with the two proposed numerical algorithms and we analyze their differences in terms of velocity, pressure and wall shear stresses (WSS) (Section 4.5). In Section 4.6 we discuss the accuracy of the Closed-0D model in comparison with the 3D-0D one. Finally, in Section 4.7 we report a comparison between a healthy and a Pulmonary Arterial Hypertension (PAH) cases.

### Numerical Experiments Setting

The numerical algorithms were implemented in life^x^,^2^ a high-performance object-oriented Finite Element library focused on the mathematical models and numerical methods for cardiac applications. It is developed in the iHEART^3^ project at the MOX Laboratory, Dipartimento di Matematica, Politecnico di Milano. The numerical simulations were run on clusters with processor Xeon E5-2640 v4 with 20 core, a base frequency of 20 GHz, and with RAM of 63 GB.

The 3D computational domain of the pulmonary arteries is reconstructed from CT scans provided by the Division of Cardiovascular Surgery of “Luigi Sacco” Hospital, Milan, by means of the Vascular Modeling ToolKit (VMTK, see [1]), which allows also to generate the corresponding hexahedral computational mesh (see Section 4.3).

We set the blood density $\rho = 1.06 \cdot 10^{3} \frac {\text {Kg}}{\mathrm {m}^{3}}$, dynamic viscosity *μ* = 3.5 ⋅ 10^3^Pa ⋅s, time step *Δt* = 0.001s and a heartbeat period *T* = 0.8s. The linear system arising after linearization and discretization is solved by means of the GMRES method with a maximum number of iterations equal to 1000 and an absolute tolerance of 10^− 10^. The backflow stabilization (see Section 3.4) is applied on every outlet boundary with *β* = 1.

In Table 1, we report all the values of the lumped parameters used in the 0D models of the two algorithms. Notice that common values used in the Open- and Closed-0D models were taken from [46]. Instead, the specific values (capitalized in the table) corresponding to the pulmonary artery in the Closed-0D model (black box in Fig. 2) were calibrated in order to maximize the accordance between the 3D-0D and the Closed-0D results during the third heartbeat. Notice that the values of the resistance, compliance and inductance of the proximal compartment are chosen in order to prevent numerical instabilities. For this reason, their values, in particular the compliance one, could have not a physical meaning and are much larger than the other ones.

**Table 1:** Resistance ($\frac {mmHg \cdot s}{ml}$), inductance ($\frac {mmHg \cdot s^{2}}{ml}$), capacity ($\frac {ml}{mmHg}$) and elastance ($\frac {mmHg}{ml}$) values of both (Open- and Closed-) 0D models

Right atrium	*E*_*a*_	0.06
	*E*_*b*_	0.07
	*T*_*contr*_	0.17
	*T*_*relax*_	0.17
Tricuspid valve	$R_{\min \limits }$	75 ⋅ 10^− 3^
	$R_{\max \limits }$	75 ⋅ 10^3^
Right ventricle	*E*_*a*_	0.55
	*E*_*b*_	0.05
	*T*_*contr*_	0.34
	*T*_*relax*_	0.15
Pulmonary valve	$R_{\min \limits }$	75 ⋅ 10^− 3^
	$R_{\max \limits }$	75 ⋅ 10^3^
Proximal compartment	*R*_*IN*_	3.21 ⋅ 10^− 2^
	*L*_*IN*_	2.50 ⋅ 10^− 3^
	*C*_*IN*_	3.90
Pulmonary artery	*R*_*PA*_	2.50 ⋅ 10^− 4^
	*L*_*PA*_	2 ⋅ 10^− 3^
	*C*_*PA*_	5 ⋅ 10^− 4^
Microvasculature and lungs	*R*_*OUT*_	2.29 ⋅ 10^− 2^
	*L*_*OUT*_	1.65 ⋅ 10^− 3^
	*C*_*OUT*_	0.25
Pulmonary venous system	$R^{PUL}_{VEN}$	3.56 ⋅ 10^− 2^
	$L^{PUL}_{VEN}$	5 ⋅ 10^− 4^
	$C^{PUL}_{VEN}$	16
Left atrium	*E*_*a*_	0.07
	*E*_*b*_	0.09
	*T*_*contr*_	0.17
	*T*_*relax*_	0.17
Mitral valve	$R_{\min \limits }$	75 ⋅ 10^− 3^
	$R_{\max \limits }$	75 ⋅ 10^3^
Left ventricle	*E*_*a*_	2.75
	*E*_*b*_	0.08
	*T*_*contr*_	0.34
	*T*_*relax*_	0.15
Aortic valve	$R_{\min \limits }$	75 ⋅ 10^− 3^
	$R_{\max \limits }$	75 ⋅ 10^3^
Systemic arterial system	$R^{SYS}_{ART}$	0.64
	$L^{SYS}_{ART}$	5 ⋅ 10^− 3^
	$C^{SYS}_{ART}$	1.2
Systemic venous system	$R^{SYS}_{VEN}$	0.26
	$L^{SYS}_{VEN}$	5 ⋅ 10^− 4^
	$C^{SYS}_{VEN}$	60

In caps the values of the pulmonary artery block, holding only for the Closed-0D model

As for the time discretization of the 0D model, we employed the explicit Euler scheme for the test reported in Section 4.2 whereas the 4th order Runge–Kutta explicit method for the other tests.

### Test I: Splitting-Explicit vs. Splitting-Implicit Algorithms

**Fig. 3:**
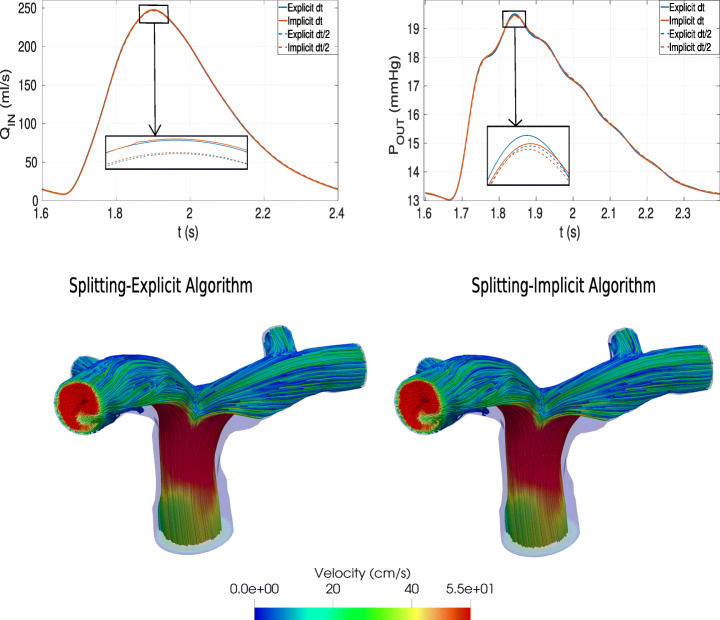
Comparison between implicit and explicit solutions. Top-Left: Inlet flux *Q*_*IN*_. Top-Right: Outlet pressure *P*_*OUT*_. Bottom: Velocity field at the systolic peak (*t* = 1.9s). Left: Splitting-explicit algorithm; Right: Splitting-implicit algorithm. Test I

In this section we want to assess a comparison between the SE and the SI algorithms. The value *ε* = 0.001 has been used as tolerance in the relative stopping criterion of Algorithm 2. In Fig. 3, we report in the first row the comparison for the interface quantities computed by the 0D model, i.e. *Q*_*IN*_ and *P*_*OUT*_. From these results we observe an excellent agreement between the explicit and implicit strategies. Moreover, we report the same comparison also for a halved time step, i.e. *Δt* = 0.0005 s. As expected from the theory, the two strategies tend to the same solution for decreasing values of *Δt*, being two different time discretization of the interface coupling. We observe that convergence of the 3D-0D coupling was achieved with an average number of iterations equal to 5.0 and 3.9 for *Δt* = 0.001 s and *Δt* = 0.0005 s, respectively.

In the second row of Fig. 3, we report the 3D velocity field on a slice at time *t* = 1.9 s (systolic peak). This result shows that, as expected, also the explicit and implicit 3D solutions are in excellent agreement. As anticipated (see Footnote 1), the results obtained with the SE algorithm were stable. We deduce that the value of *Δt* = 0.001 s selected for our simulations is below the constraint required by the explicit treatment. Thus, since SE algorithm reveals to be also accurate, in what follows we consider only the Splitting-Explicit algorithm with *Δt* = 0.001 s.

### Test II: Mesh Convergence

We carry out a mesh convergence study to investigate the accuracy of our numerical solution by refining the grid. To this aim, we consider here three grids having a different space step discretization, namely:

- Fine grid, *h*_1_ = 0.4 mm,
- Medium grid, *h*_2_ = 0.7 mm,
- Coarse grid, *h*_3_ = 1.1 mm.

It is worth noting that the space discretization steps have a constant ratio, $r = \frac {h_{2}}{h_{1}} = \frac {h_{3}}{h_{2}} = 1.6$. On a longitudinal slice, obtained by cutting the 3D pulmonary artery, we compute the integrals *f*_*i*_, *i* = 1,2,3, of the pressure field for the three grids, used as indices for the convergence analysis. Then, we estimate the order of convergence (*p*), the constant of the numerical method (*c*) and the reference solution (*f*_*ref*_), by means of the Richardson extrapolation [37],

$$ p = \frac{\log\left( \frac{f_{3}-f_{2}}{f_{2}-f_{1}}\right)}{\log(r)}, \quad f_{ref} = \frac{({h_{2}^{p}} \cdot f_{3}-{h_{3}^{p}} \cdot f_{2})}{(-{h_{3}^{p}} + {h_{2}^{p}})}. $$

Finally, we compute the relative discrepancies among the meshes as follows:

$$ E_{i} = \frac{|f_{i} - f_{ref}|}{|f_{ref}|}, \qquad i=1,2,3. $$

Given the value of *r* used in this analysis, an acceptable value for the relative discrepancy under which we can argue that the solution has reached convergence, is 3%.

**Fig. 4:**
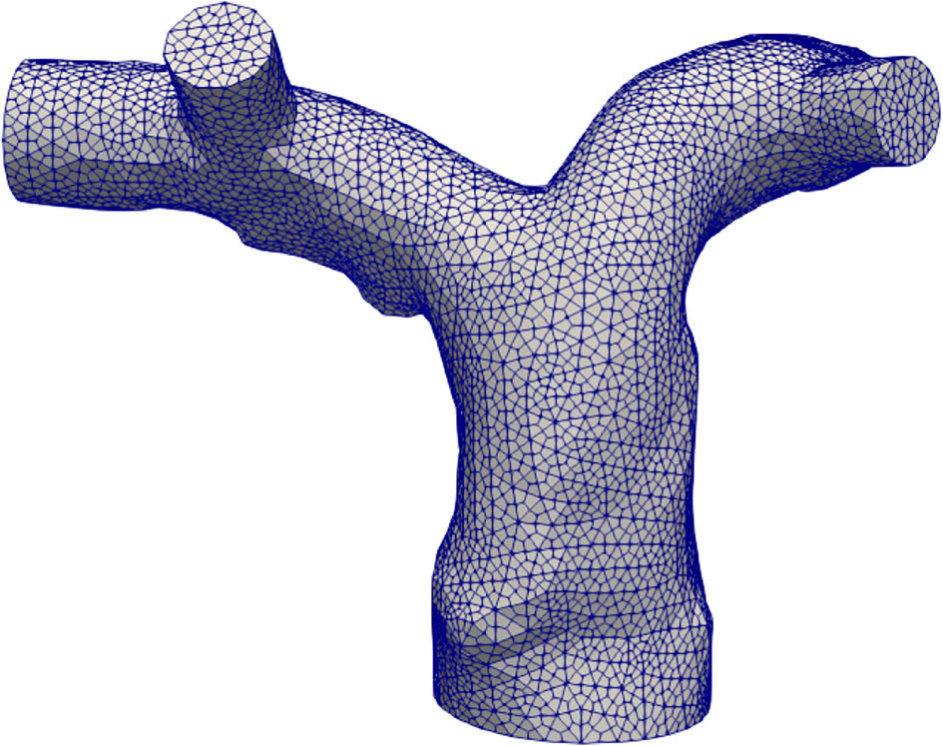
Medium grid of the patient-specific pulmonary artery used for all the numerical tests, anterior view. Test II

In Table 2, we report the quantitative values used to perform the mesh convergence. From these results, we find that the medium mesh (average cell size *h* = 0.7 mm, corresponding to 84992 cells, see Fig. 4), satisfies the convergence requirement, namely, a relative error less than 3%. Thus, we decide to use it for the all the numerical simulations.

**Table 2:** For the three different meshes: total number of grid cells *N*, index *f*_*i*_ used for the convergence analysis, relative discrepancy *E*_*i*_

Mesh	N	*f*_*i*_	*E*_*i*_(*%*)
Coarse	26692	6.754	4.9
Medium	84992	6.774	2.9
Fine	298176	6.785	1.8

Mesh convergence. Test II

It is worth noting that the simulations for the grid convergence were done with a fixed time step of 10^− 3^ s that guaranteed that the temporal error was below the spatial one.

### Test III: About the Stability of Coupling Algorithms

In Section 2 we have discussed the introduction of a RLC model (the red one in Fig. 1) between the 3D pulmonary artery and the pulmonary valve (downstream diode in the light blue block). This RLC model represents the first 0.3cm of the pulmonary artery and allows to avoid the direct connection of the 3D model with the diode, which, as detailed below for the first time, may lead to unstable results. This lumped parameter model must be suitably devised to guarantee a correct mathematical transition between the models.

**Fig. 5:**
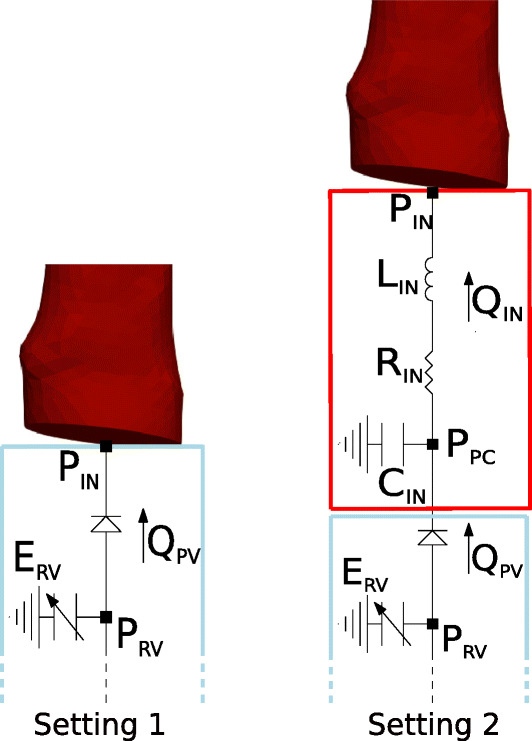
Left: The 3D model is directly connected with the pulmonary valve (Setting 1). Right: The 3D model is connected with the complete Open-0D models (Setting 2). Test III

In order to valuate the effect of this RLC model on the Splitting-Explicit and One-Way algorithms, we considered two scenarios: the first one where in the Open-0D model such block is eliminated and the 3D model is connected directly with the pulmonary valve diode (light blue block); we refer to this scenario as Setting 1, see Fig. 5, left; and a second one where the 3D model is connected to 0D pulmonary valve through the proximal RLC compartment (Setting 2, see Fig. 5, right).

For the sake of completeness, we report in what follows the terms of the equations differentiating the two Settings:

*Setting 1* Pulmonary valve

$$ Q_{PV}= \frac{P_{RV}-P_{IN}}{R_{PV}(P_{RV},P_{IN})}. $$

*Setting 2* Pulmonary valve

$$ Q_{PV}= \frac{P_{RV}-P_{PC}}{R_{PV}(P_{RV},P_{PC})}. $$

Proximal compartment

$$ \begin{array}{@{}rcl@{}} C_{IN}\frac{dP_{PC}}{dt}&=& Q_{PV}-Q_{IN},\\ L_{IN}\frac{dQ_{IN}}{dt}&=& -R_{IN}Q_{IN}-(P_{IN}-P_{PC}). \end{array} $$

**Fig. 6:**
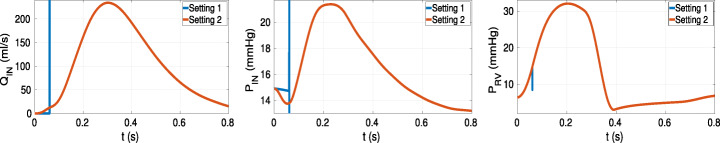
Comparison between the results obtained with Setting 1 (blue) and Setting 2 (red). Left: 0D inlet flow rate (*Q*_*IN*_). Center: 3D inlet mean pressure (*P*_*IN*_). Right: 0D right ventricular pressure (*P*_*RV*_). Test III

In Fig. 6 we report, for the Splitting-Explicit Algorithm, the inlet quantities at the first heartbeat together with the ventricular pressure computed by the 0D model in both Settings 1 and 2. Similar results are obtained for the One-Way Algorithm. In particular, the inlet flow rate *Q*_*IN*_ is computed by the 0D model whereas the inlet mean pressure *P*_*IN*_ by the 3D model. We can observe unstable solutions just after the first time steps in the case of Setting 1. The arising of such instabilities seems to be independent of the choice of the time step *Δt*. Moreover, varying the resistance of the non-ideal diode modeling the pulmonary valve does not introduce any improvement. Instead, the complete Open-0D model accounting also for the RLC (red block) lumped model (Setting 2, see Fig. 5, right), allows to get stable results. We notice a small, non-physiological pressure drop during the first time instants. This is due to the fact that the solution is not completely developed and has not yet reached a regime state. This pressure drop disappears during the following heartbeats as clearly showed in Fig. 9.

We also observed a significant difference (about 10 mmHg) between *P*_*IN*_ and *P*_*RV*_, that is the pressure at the inlet of the 3D model and the 0D ventricular pressure, respectively. This is equivalent to the pressure drop across the RLC block, see Fig. 5, right. Thus, the price to pay to have stability is the formation of a large pressure drop corresponding to a short tract of the pulmonary artery (the RLC block), which seems to be larger than expected. The instability behaviour reported above could be explained by observing that in Splitting-Explicit Algorithm for 3D-0D coupling the concept of *bridging regions* plays a fundamental role [7, 26, 33]. In particular, when the 3D model gives to the Open-0D model an information about the pressure, the latter becomes a forcing term for the Open-0D model making necessary the presence of an inductive term at the interface to allow the calculation of the flow rate representing a state variable for the Open-0D model. On the other hand, if the 3D model gives an information about the flow rate to the Open-0D model, a compliance must be present at the interface, in order to calculate the pressure representing a state variable for the Open-0D model. Thus, the direct connection of the 3D model with the diode does not guarantee, for both Splitting-Explicit and One-Way Algorithms, the satisfaction of such principle and this explains the unstable solutions.

### Test IV: Comparison between Coupling Algorithms

**Fig. 7:**
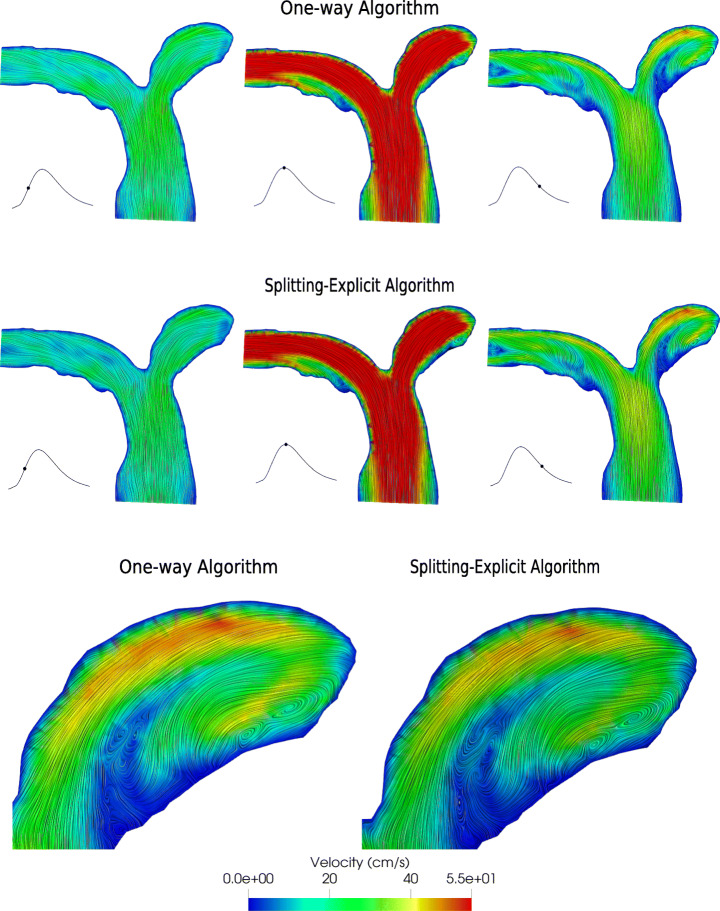
Top: Velocity field at three different istants for the One-way and Splitting-Explicit algorithms. Left: Acceleration phase (*t* = 1.75 s). Center: Systolic peak (*t* = 1.9 s). Right: Deceleration phase (*t* = 2.15 s). Bottom: Zoom on the right pulmonary artery during the diastolic phase. Test IV

In this section, we report and discuss the results obtained by means of the two coupling algorithms introduced in Section 3, namely the Splitting-Explicit Algorithm and the One-Way Algorithm. In particular, in Fig. 7 we report for a longitudinal slice the comparison between velocity fields at three different temporal instants of the heartbeat, namely the acceleration phase (*t* = 1.75 s), the systolic peak (*t* = 1.9 s) and the deceleration phase (*t* = 2.15 s). The results do not present any relevant difference in terms of flow pattern and magnitude. We observe a recirculation region in the inferior side of the right pulmonary artery during the systolic peak. Some vortices are generated during the deceleration phase, which are slightly different in the two cases. In Fig. 7, we also report a zoom on the streamlines of the right pulmonary artery during the diastolic phase.

**Fig. 8:**
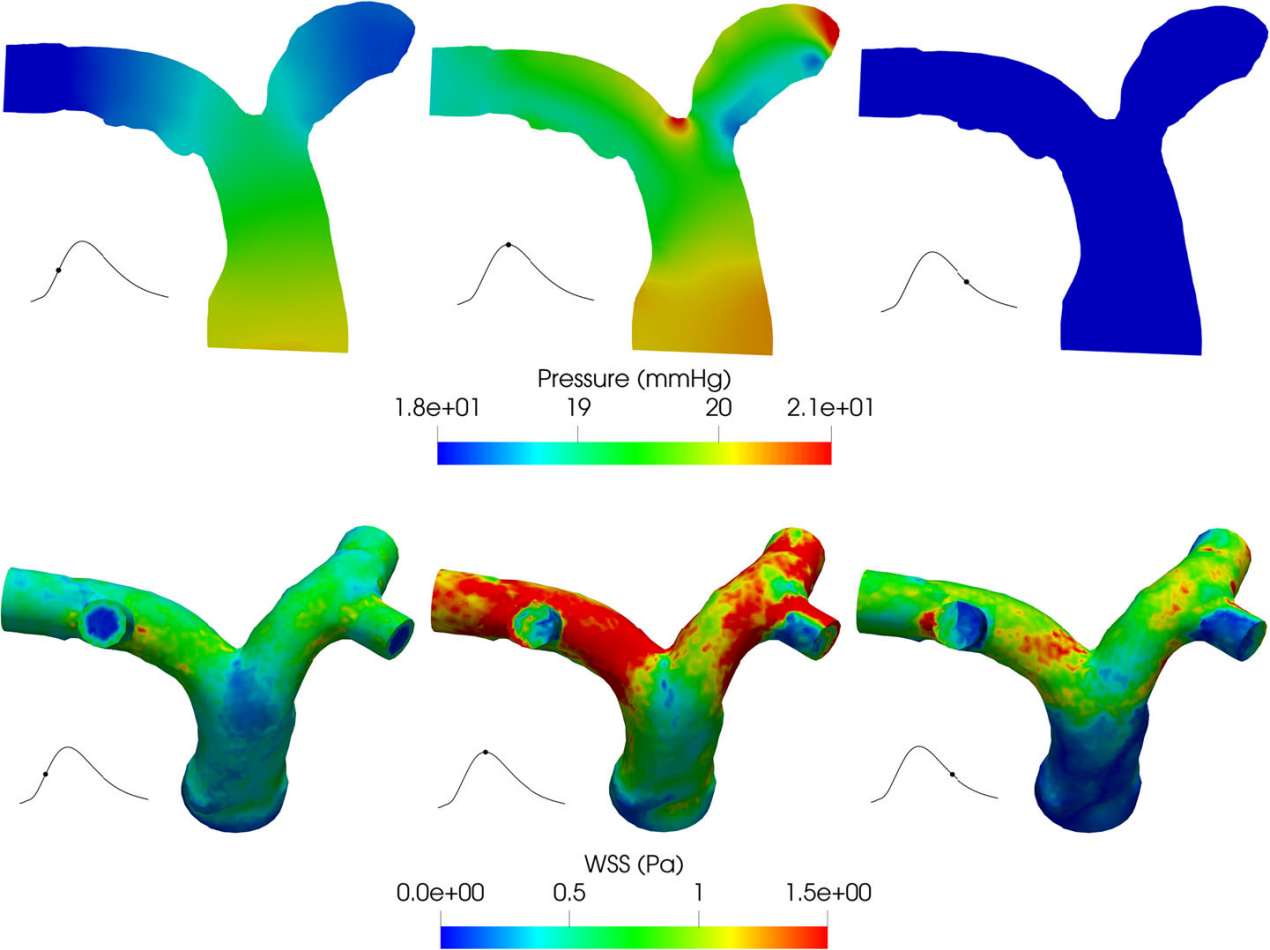
Pressure field (top) and WSS (bottom) for the Splitting-Explicit algorithm. Left: Acceleration phase (*t* = 1.75 s). Center: Systolic peak (*t* = 1.9 s). Right: Deceleration phase (*t* = 2.15 s). Test IV

On the same section, we report in Fig. 8 the pressure field obtained with the Splitting-Explicit Algorithm. The solution of the One-Way Algorithm is almost identical, thus it is not shown. At the systolic peak, a high pressure is experienced at the inlet.

On the branching of the left and right pulmonary arteries, there is a stagnation point that generates the highest pressure of the whole pulmonary arteries. Finally, in the second row of Fig. 8, we show the WSS field on the physical wall, again only for the Splitting-Explicit algorithm since relevant differences are not found between the two algorithms. WSS measures the tangential viscous stress exerted by the blood in motion onto the vessel walls [12]:

$$ WSS = \|\boldsymbol{T}\boldsymbol{n} - (\boldsymbol{T} \boldsymbol{n} \boldsymbol{n})\boldsymbol{n}\|, $$

where ***T*** represents the Cauchy stress tensor. In particular, the WSS is reported from the anterior view in order to highlight the differences between the main and distal branches. The WSS magnitude is strongly related to the velocity, therefore we see a higher WSS in the distal branches of the pulmonary vasculature where the diameter of the vessel decreases.

The results obtained with this test show that the proposed method is able to simulate the hemodynamics of the pulmonary arteries obtaining physiological results [22, 42]; the pulmonary arterial pressure is between 14–20 mmHg, the WSS is between 0–3 Pa and the right ventricular volume is between 60–160 ml.

### Test V: About the Accuracy of the Closed-0D Model

**Fig. 9:**
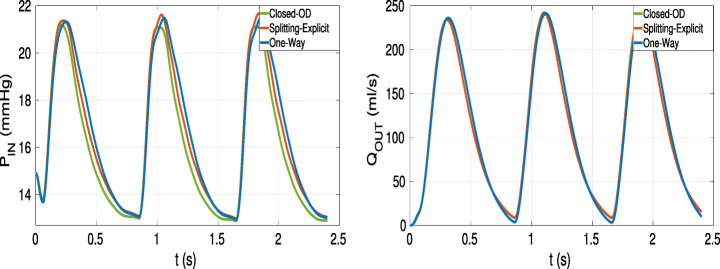
Interface quantites. Left: Inlet pressure (*P*_*IN*_). Right: Outlet flow rate (*Q*_*OUT*_). Test V

The Closed-0D model (i.e the model where the 3D pulmonary artery is substituted with a 0D model, black box in Fig. 2) allows the calculation of the mean pressure and flow rate in the whole cardiovascular system with a convenient computational cost; therefore, it is a powerful framework that can give a quantitative indication about the haemodynamics at least in terms of averaged flow properties as mean pressures and flow rates. In Fig. 9, we compare the mean pressure *P*_*IN*_ and the flow rate *Q*_*OUT*_ inside the pulmonary artery compartment computed by the Closed-0D model with those computed by means of the 3D model for both the Splitting-Explicit and One-Way Algorithms.

From these results, we notice that the Closed-0D model, properly calibrated (see Table 1), is able to give accurate results able to capture almost perfectly the mean quantities, i.e the inlet mean pressure and the outlet flow rate. In the flow rate, we are not able to see any difference, instead in the pressure field, the models present the bigger differences at the systolic peak and during the deceleration phase. Of course, to have a fully detailed description of the blood flow, as the local pressure, velocity field and WSS, a 3D-0D coupled model is needed (see Fig. 2).

### Test VI: Comparison between Healthy and Pulmonary Arterial Hypertension Cases

**Fig. 10:**
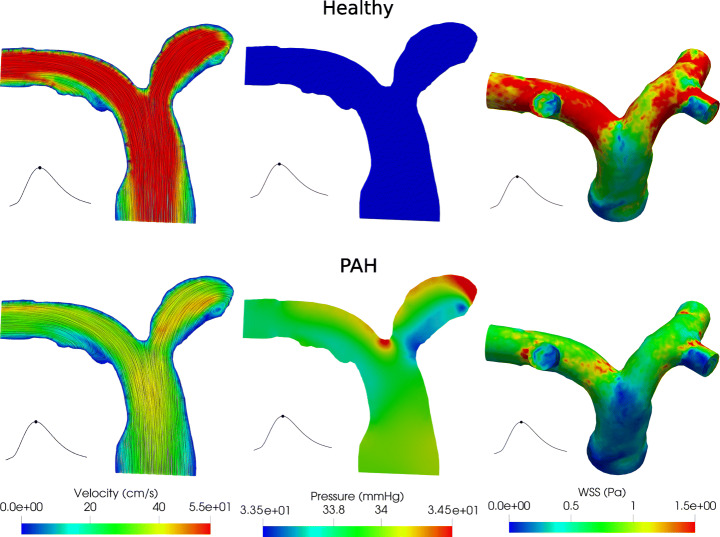
Systolic peak (*t* = 1.9 s). Left: Velocity field. Center: Pressure field. Right: WSS field. Test VI

In this final test, we compare the hemodynamics in the pulmonary artery in the healthy and PAH diseases. To this aim, we use the Splitting-Explicit Algorithm. The PAH is a disease characterized by an elevated resistance in the distal branches of the pulmonary arteries (the microvasculature and the lungs compartment); this condition entails an increase in the working pressure of the right ventricle, possibly causing hypertrophy and failure [19]. It has been demonstrated that the hemodynamics parameters are significant indicators of PAH; in particular, changes in the right ventricle end-diastolic volume and a decrease of WSS at the proximal part of the pulmonary artery are suggested as a good indicator of PAH severity [45]. We model the PAH disease by increasing the resistance of microvascolature and lungs compartment: specifically, we quintuplicate the *R*_*OUT*_ value with respect to the physiological case choosing $R_{OUT} = 1.14 \cdot 10^{-1} \frac {\textrm {mmHg} \cdot \textit {s}}{\text {ml}}$. Using the same visualizations of Figs. 7 and 8, in Fig. 10 we compare the velocity, pressure and WSS fields of the healthy and simulated PAH cases at the systolic peak (*t* = 1.9 s).

From these results, we observe that the PAH is characterized by a strong decrease in term of velocity due to the resistance increase of the 0D microvasculature and lungs compartment representing physically the distal branches of the pulmonary arteries. This increase leads to a higher pressure along the whole pulmonary artery, and consequently to lower velocities. Since the WSS is strongly related with the velocity field, we find that the PAH is also associated with a lower WSS than the healthy case, specially in the distal branches. In particular, we observe a decrease of about 40% of the WSS. It is worth noting that in the PAH disease, the pressure reached at the systolic peak is about 34 mmHg, almost 1.5 times more than the pressure reached in the healthy case. As a confirmation of such results, in Table 3, we report the time-averaged values (computed during the third heartbeat) of the inlet pressure $P^{MEAN}_{IN}$ and flow rate $Q^{MEAN}_{IN}$ for both the scenarios. Moreover, we report also the time average right ventricle pressure $P^{MEAN}_{RV}$ and end diastolic right ventricle volume $V^{MEAN}_{RV}$.^4^ From these results, we observe that for the PAH case, as expected, also $P^{MEAN}_{RV}$ increases. Interestingly, we also observe that the end-diastolic volume increases of about 10% in the PAH case. This could be ascribed to Frank-Starling law, which allows the ventricle to increase its volume in order to win the increased resistances.

**Table 3:** Averaged in time quantities for the healthy and PAH cases. Test VI

Case	$Q^{MEAN}_{IN}$ (ml/s)	$P^{MEAN}_{IN}$ (mmHg)	$P^{MEAN}_{RV}$ (mmHg)	$V^{MEAN}_{RV}$ (ml)	SV (ml)
Healthy	111.1	16.4	13.4	154.4	88
PAH	97.3	27.7	16.2	166.1	82

**Fig. 11:**
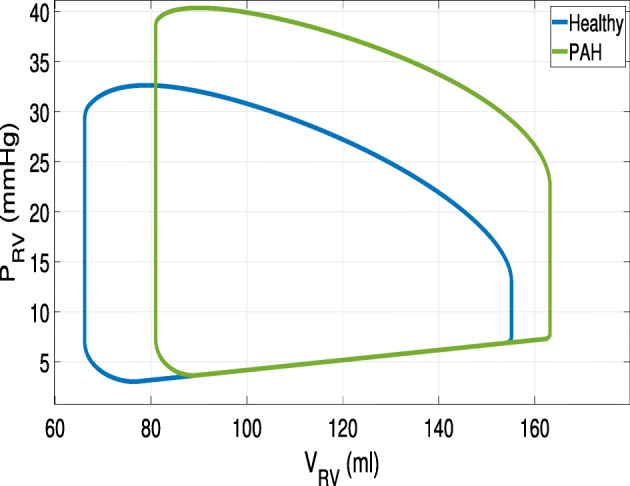
Pressure-volume loop of the right ventricle. Test VI

Moreover, we report in Fig. 11, the right ventricle pressure-volume loop of both the scenarios. According to the literature the PAH case is characterized by an increase of pressures and volumes [19, 42, 45], corresponding to a decrease of the stroke volume.

## Conclusions and Limitations

We have simulated the hemodynamics of the pulmonary arteries in a geometric multiscale context. Physiological inlet and outlet boundary conditions are provided to the 3D model of the pulmonary arteries by means of a lumped parameter model of the entire cardiovascular system.

We adopted three strategies for the coupling of the 3D and 0D models: the One-Way, the Splitting-Explicit and the Splitting-Implicit algorithms. Numerical results have demonstrated that all these algorithms are able to reproduce physiological results in accordance with the literature. The algorithms provide substantially equivalent results in terms of velocity and WSS, and some slight differences for the pressure.

We have also simulated PAH disease increasing the resistance of the microvasculature and lungs compartment. We found that, as expected, PAH produces a strong increase of the pressures with respect to the healthy case and consequently, lower velocities and WSS, in particular in the distal branches.

This study presents some limitations. First, the 3D pulmonary arteries were modeled by means of the rigid walls assumption. This is a restrictive choice because it leads to an overestimation of pressure, velocity and WSS, especially in the distal branches, as found in [23]; for a simulation including the 3D model of the microvasculature, the fluid-structure interaction approach becomes mandatory. Moreover, the pulmonary arterial stiffness seems to be one of the principal biomechanical markers for the identification of PAH disease [45], therefore an FSI simulation may give more accurate information about PAH.

Second, the lumped parameter model of the cardiovascular system is relatively simple; other RLC compartments could be added in order to consider different parts of the cardiovascular system.

Third, we have assumed the same outlet pressure at the four distal pulmonary outlets. This is an approximation, since some pressure differences (although small) may appear and in any case the length of the branches is not identical. Different RLC compartments should be considered for each outlet. However, since this work is focused on comparison among different algorithms and scenarios (all affected by this limitation), we believe that our assumption may be in first approximation acceptable.

In addition, both the algorithms have practically the same computational time taking about 24 hours to simulate three heartbeats of 0.8s as period using the medium grid; moreover, no relevant computational time differences between the healthy and the PAH case have been observed.

## Footnotes

1 Notice however that in our numerical simulations we did not find any constraint for *Δ**t* when using the SE algorithm, meaning that its choices, driven by accuracy purposes, satisfy the stability bound. See Section 4

2 https://lifex.gitlab.io/

3 iHEART - An Integrated Heart model for the simulation of the cardiac function. European Research Council (ERC) grant agreement No 740132.

4 Remember that $P^{MEAN}_{IN}$ and $Q^{MEAN}_{IN}$ are computed from the 3D simulation, whereas $P^{MEAN}_{RV}$ and $V^{MEAN}_{RV}$ from the 0D model.
